# Immunogold Localization of Vitellogenin in the Ovaries, Hypopharyngeal Glands and Head Fat Bodies of Honeybee Workers, *Apis Mellifera*

**DOI:** 10.1673/031.007.5201

**Published:** 2007-10-25

**Authors:** Siri-Christine Seehuus, Kari Norberg, Trygve Krekling, Kim Fondrk, Gro V. Amdam

**Affiliations:** ^1^ Department of Animal and Aquacultural Sciences, Norwegian University of Life Sciences, P. O. Box 5003, N–1432 Aas, Norway; ^2^ Department of Plant- and Environmental Sciences, IPM-Microscopy, Norwegian University of Life Sciences, P. O. Box 5003, N-1432 Aas Norway; ^3^ School of Life Sciences, Arizona State University, Tempe, AZ 85287, U.S.A

**Keywords:** immunohistochemistry, anti-vitellogenin, oocyte, trophocyte, secretory acini, adipose tissue, vesicle transport, aging

## Abstract

Vitellogenin is a yolk precursor protein in most oviparous females. In the advanced eusocial honeybee, *Apis mellifera* (Hymenoptera: Apidae), vitellogenin has recently attracted much interest as this protein, in addition to a classical function in oocyte development in the reproductive queen caste, has evolved functions in the facultatively sterile female worker caste not documented in other species. However, research on the spatial dynamics of vitellogenin in various tissues is not easily performed with available tools. Here we present an immunogold staining procedure that visualizes honeybee vitellogenin in resin embedded tissue. To establish the protocol, we used ovaries of worker bees from colonies with and without a queen. Under the first condition, vitellogenin is assumed not to be present in the workers' ovaries. Under the second condition, the ovaries of worker bees become vitellogenic, with abundant opportunities for detection of complex patterns of vitellogenin uptake and storage. By use of this experimental setup, the staining method is shown to be both sensitive and specific. To demonstrate the functional significance of the protocol, it was subsequently used to identify vitellogenin protein in the hypopharyngeal glands (brood food producing head glands) of nursing worker bees and in adjacent head fat body cells for the first time. Localization of vitellogenin in these tissues supports previously hypothesized roles of vitellogenin in social behavior. This protocol thus provides deeper insights into the functions of vitellogenin in the honeybee.

## Introduction

In insects, yolk precursor proteins are synthesized and secreted from the fat body, which is analogous to the mammalian adipose tissue and liver (Giorgi et al. 1989; Haunerland and Shirk 1995). Circulating yolk proteins are subsequently imported into growing oocytes via receptor mediated endocytosis (Telfer 1965; Fleig 1995; Tanaka and Hartfelder 2004). The honeybee (*Apis mellifera*) has only one yolk precursor protein, which is a very high-density lipoprotein with a molecular weight of 180 kDa (Wheeler and Kawooya 1990; Piulachs et al. 2003). The yolk precursor, vitellogenin, is a prerequisite for honeybee egg development (Lin et al. 1999), but has also been exploited during honeybee eusocial evolution to become an important component of pathways that are not directly linked to reproduction (Amdam et al. 2003, 2004b; Seehuus et al. 2006a).

The honeybee is an advanced eusocial organism characterized by differentiation of female forms into two distinct phenotypes; the long-lived and highly fecund queen, and the facultatively sterile worker (Winston 1987). Workers further differentiate into temporal stages that perform different tasks (Seeley 1982). The “nurse bee” phenotype works in the central nest and cares for brood (eggs, larvae and pupae). After 2–3 weeks, nurse bees make a transition to the forager stage and become responsible for collecting nectar, pollen, water and propolis in the field. A third phenotype develops in temperate climates during periods without brood rearing. This stress-resistant (Seehuus et al. 2006a) and long-lived worker form (lifespan up to 280 days, Sekiguchi and Sakagami 1966) is referred to as the “diutinus stage” or “winter bee” phenotype (Maurizio 1950; Fluri et al. 1982; Omholt and Amdam 2004). Diutinus bees stay in the nest and survive unfavorable periods such as winter (Maurizio 1950; Huang and Robinson 1995). They differentiate into nurse bees and foragers when conditions improve and brood rearing commences (Maurizio 1954; Sekiguchi and Sakagami 1966).

Interestingly, vitellogenin is not only produced by the honeybee queen, but is also synthesized at high levels in the nurse bees (Engels 1968). The protein further accumulates in the hemolymph of diutinus workers (as exemplified by winter bees in late fall, see above) (Fluri et al. 1982). These patterns were only recently understood when it was determined that vitellogenin is used by nursing workers in production of brood food (Amdam et al. 2003), and that the protein also can slow aging in workers and queens by scavenging free radicals and thereby increasing resistance against oxidative stress (Seehuus et al. 2006a). Furthermore, vitellogenin gene activity has been shown to suppress the circulating level of the systemic hormone juvenile hormone, which typically increases at onset of foraging behavior in workers (Guidugli et al. 2005). This finding has led to the hypothesis that vitellogenin and juvenile hormone together influence worker social behavior by being part of the endocrine mechanisms that control the transition from nurse tasks to foraging activity. Thereby, vitellogenin action is implied, but not verified, in honeybee tissues other than the ovary: e.g., the hypopharyngeal glands (Amdam et al. 2003), the abdominal fat body (Amdam and Omholt 2002), and in association with the brain and neurosecretory cells (Guidugli et al. 2005).

Vitellogenin has been thoroughly studied in the context of oocyte maturation (Engels 1973; Engels 1974; Varriale et al. 1988; Hirai et al. 1998). The recent data from the honeybee, however, implies that this focus has to be expanded. Visualization techniques have contributed to substantial insights into the roles and functions of other proteins and peptides; for instance in stress signaling pathways (Segui-Simarro et al. 2005), in patterns of carbonylation damage (Jana et al. 2002; Seehuus et al. 2006b), and in dynamics of cellular localization (Hwangbo et al. 2004). Methods for visualization of vitellogenin in tissue may therefore contribute to a deeper understanding of vitellogenin action in honeybees. In this study, we took advantage of the well-established knowledge of oocyte maturation to validate an immunogold immunohistochemical staining procedure for honeybee vitellogenin protein. Using worker ovaries as the substrate, we show that a high resolution method can accurately document vitellogenin transport and storage. After initial validation, the protocol was used to show for the first time that vitellogenin protein is found in the central duct and secretory acini of the brood food producing head glands of nurse bees (hypopharyngeal glands) and also in the worker head fat body close to the brain. These results provide initial evidence for the hypothesis that vitellogenin is utilized for brood food production, as suggested by Amdam and co-workers (2003). The method creates opportunities for future studies of the proposed role of vitellogenin protein in affecting behavior at the intersection between the head fat body, brain and neurosecretory tissues (Amdam and Omholt 2003; Hunt *et al*. 2006).

## Materials and Methods

### Antibody against 180 kDa vitellogenin

A polyclonal antibody against the 180 kDa vitellogenin protein was raised in rabbits by the Norwegian Institute of Public Health (Oslo, Norway). Eggs were gathered and pooled, 800 eggs per ml 50 mM Tris buffer, pH= 7.1. The samples were sonicated, homogenized and boiled for 2 minutes under non-reducing conditions (without DTT) before they were separated on 7% SDS-PAGE gels. Gels were stained briefly with Servablau W (0.1% in destilled-H_2_O) before the 180 kDa vitellogenin band (Wheeler and Kawooya 1990) was cut out. The gel pieces were homogenized in phosphate buffered saline solution (PBS) and the homogenate frozen for shipment to the Norwegian Institute of Public Health. Two rabbits were injected with 0.5 mg antigen diluted in 1 ml PBS and emulgated with equal amounts of Freunds Complete Adjuvant. After 17 days, the rabbits were re-immunized with 200 μg antigen diluted in PBS and emulgated with Freunds Incomplete Adjuvant. Subsequently, rabbits were boosted one time per month (two times) with 200 μg antigen diluted in PBS with Freunds Incomplete Adjuvant. Blood serum was extracted and tested for antibody titer via Western blots, using egg homogenate and hemolymph from nurse bees as positive controls. Specificity was compared to established antibodies for vitellogenin described previously (Pinto et al. 2000).

### Honeybees

To obtain samples of ovaries for immunohistochemistry, workers were marked on the thorax with a spot of paint (Testors Enamel) at adult emergence and introduced into colonies with and without a queen in an apiary at University of California, Davis. After 10–15 days, marked workers were collected and anesthetized on ice before ovaries were dissected and classified according to ovarian developmental stage: 1, previtellogenic non-activated ovary; 2, previtellogenic activated ovary; 3, vitellogenic ovary with developing oocytes; 4, mature ovary with at least one egg, as previously described by Hartfelder and co-workers (2002) and modified by Amdam and co-workers (2006). Ovaries for immunohistochemistry were stored at 4° C in 0.1 M HEPES buffer (Sigma-Aldrich) until further processing.

To obtain samples of head tissues including hypopharyngeal glands for immunohistochemistry, newly emerged workers were marked with a spot of paint and introduced into colonies with a queen in the apiary of the Norwegian University of Life Sciences, Aas. After 8 days, marked workers presumably engaged in nurse tasks were collected by gathering marked bees with their heads and thoraxes in cells containing larvae. The heads of the bees were stored at 4°C in 0.1 M HEPES buffer (Sigma-Aldrich, www.sigmaaldrich.com) until further processing.

To obtain samples for independent Western blot verification of the general findings, ovary as well as fat body tissues from the abdomen and head of queens, which are documented to be particularly abundant in vitellogenin (Engels 1972,^1974^, Fluri et al. 1981, Engels et al. 1990) were obtained from 1 year-old egg-laying queens (Weaver Apiaries, Texas). Hypopharyngeal glands were harvested from nurse bees (assumed to be vitellogenin positive) and foragers (assumed to be vitellogenin negative) in the apiary of Arizona State University, Phoenix Arizona. Nurse bees (with head and thorax in a cell containing larvae) were collected from the central brood area of colonies with a queen. Foragers (returning to the colonies with pollen loads) were retrieved at the hive entrances. Queens and workers were anesthetized on ice before tissues were dissected and stored in Tris-buffer (20 mM Tris-HCl, 150 mM NaCl, 5mM EDTA), pH 7.5, supplemented with proteinase inhibitors (Amdam et al. 2004a) until further processing.

### Immunohistochemistry

Ovaries and heads were fixed in 4% formaldehyde solution (methanol free, Polysciences, www.polysciences.com) supplemented with 0.2% Triton X-100 for 12 hours at room temperature. Fixed tissue was thoroughly washed in 0.1 M PIPES buffer (Sigma-Aldrich) before dehydration in an ethanol series (70%, 90%, 95%, and 4 × 100%), each step for 15 min. After dehydration the tissues were infiltrated with a graded series of London Resin-White (LR-White; Electron Microscopy Sciences, www.emsdiasum.com) and polymerized over night at 6o°C. For conventional light microscopy and immuno labeling, semi-thin sections (1–2 μm) were cut with a diamond knife on an ultramicrotome (Reichert ultracut E, Reichert-Jung, www.leica-microsystems.com) and dried onto SuperFrost©Plus slides (Electron Microscopy Sciences, www.emdiasum.com).

**Figure 1.**
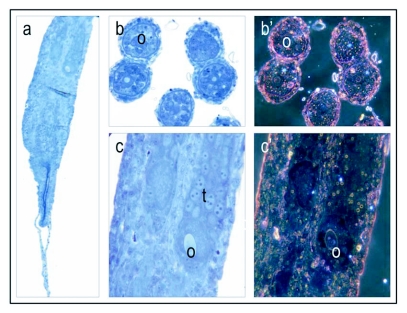
Vitellogenin staining of a stage 1, previtellogenic non-activated ovary. The low magnification micrograph (5 ×) of ovarioles (a), consists of undeveloped cells in the anterior end and more developed oogonia (0) and trophocytes (t) (nurse cells) in the posterior end. Cross sections (bright field: b and dark field b') and longitudinal sections (bright field: c and dark field: c') at a primary magnification of 40 x; show oogonia and developing multinuclear trophocytes. The ovary shows no positive staining for vitellogenin (a-c). Positive staining is shown in Figures 2–6.

**Figure 2.**
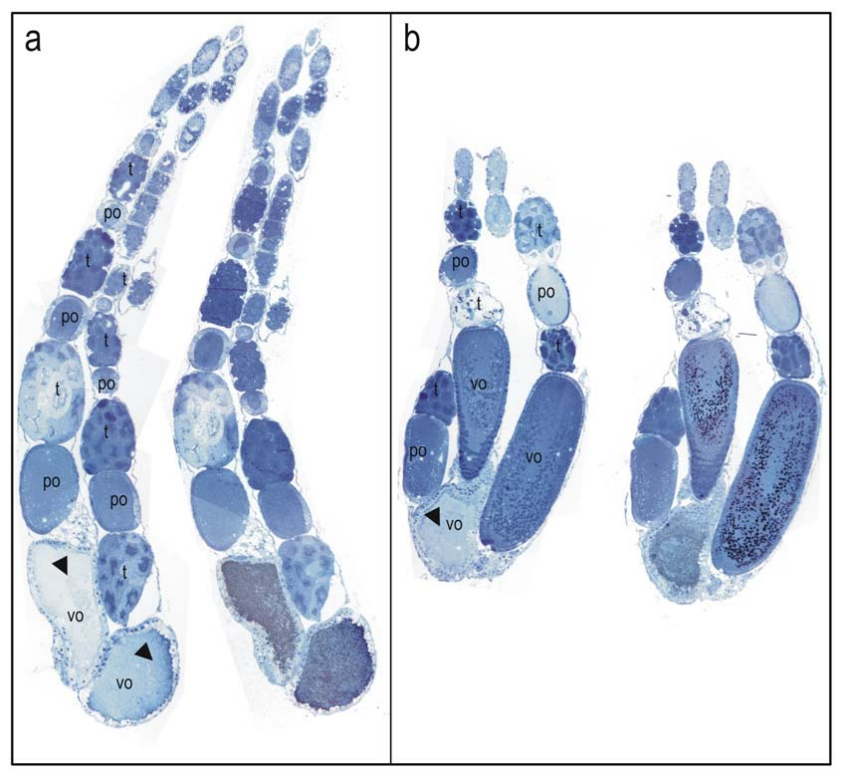
Vitellogenin staining of a stage 3, vitellogenic ovary with developing oocytes. In a and b, the immunogold stained ovary (right) with corresponding control (left) is recorded at 16 x. The images consist of a mosaic of pictures. In controls, primary antibody was substituted with buffer. The silver enhanced immunogold staining of vitellogenin shows as black dots in bright field pictures. At 16 × magnifications the separate black dots are not easily detected and appear as a black or grayish area of stain. The ovarioles consists of clusters of trophocytes (t) connected to one oocyte alternately in anterior-posterior direction. The vitellogenic oocytes (vo) appear to incorporate proteins in granules and are intensely labeled for vitellogenin (b). Some oocytes have irregular shape and may be in process of reabsorption (indicated by arrows in a and b). Abbreviations in the controls.

**Figure 3.**
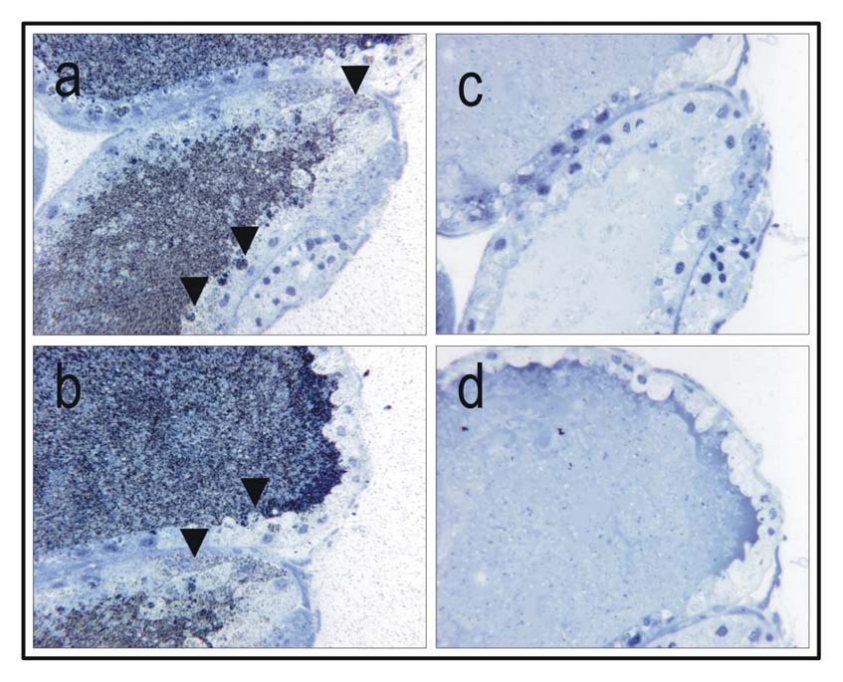
Immunolocalization of vitellogenin in a stage 3, vitellogenic oocyte; primary magnification of 40 × (a-b, corresponding controls (primary antibody substituted with buffer) c-d). The inside of the vitellogenic oocyte is covered in a grayish area of labeled antigen. The arrows point to areas where vitellogenin (gray or black dots) seems to be transported through pores in the follicle cells (a-b) or directly through the follicle cells in granules to the perivitelline space (see arrows) and thus, seems to be supporting already existing data (Fleig 1995). The controls (c-d) show no positive staining for vitellogenin.

**Figure 4.**
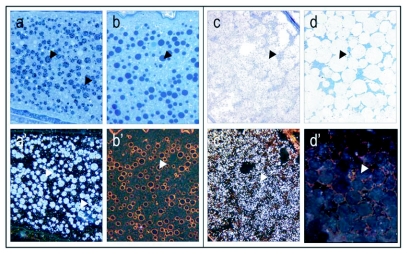
Yolk bodies in vitellogenic oocytes at recorded at 40 × magnification, (see arrows; bright field: a-d, dark field: a'-d'). Controls (b, b' and d, d') are treated with preimmune sera instead of primary antibody. At the onset of the vitellogenic phase, vitellogenin positive yolk bodies (see arrows) are numerous, small and dispersed through the oocyte (a, a'). Yolk bodies are intensely labeled against vitellogenin, whereas the areas between the yolk bodies show much lower levels of labeling. Mature oocytes, like the one depicted from a stage 4 ovary (mature ovary with at least one egg), are characterized by large vitellogenin positive globules (see arrows) that evolve from yolk body fusion (c, c'). Note absence of labeling in controls (b,b' and d, d').

**Figure 5.**
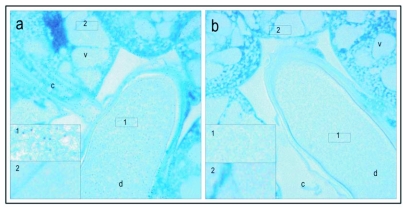
Verification of presence of mature vitellogenin protein in hypopharyngeal glands of a honeybee worker that performed brood care. Immuno labeling shows that the vacuoles (v), the canal (c) and the common duct (d), are all vitellogenin positive. The antigen is scattered in the gland and in comparison with oocytes there seems to be no aggregation of vitellogenin. In the high resolution micrograph (100 x) (a) labeling is seen as black dots (as exemplified by inserted box 1, higher magnification of stain in the common duct). The antigen is not present in all the vacuoles in a gland (see inserted box 2 in (a), higher magnification of labeling free area in the vacuole). The corresponding preimmune controls (b) show no positive labeling for vitellogenin, neither in the common duct (see inserted box 1 in (b) for higher magnification of the area) or the vacuoles (see inserted box 2 in (b) for higher magnification of the area).

For immuno labeling, sections were rinsed in 4 × 15 min with PBS supplemented with 1% bovine serum albumin (BSA, Sigma Aldrich), etched with 0.2% H_2_O_2_ in methanol, washed 4 × 15 min with PBS/1% BSA, blocked with PBS/2% BSA for one hour at room temperature, washed and incubated with a polyclonal antibody against vitellogenin at 4°C over night. Control sections were incubated with buffer only (Fig. 1–3) or with preimmune sera (Fig. 4–5 and 7). Controls treated with buffer or with preimmune sera were negative throughout the experiments, fulfilling requirements for control of antibody staining. All sections were washed 4 × 15 min with PBS and incubated with secondary antibody protein A gold (auroprobe EM prot A G10, Amersham Biosciences, www.apbiotech.com) over night at 4°C. The sections were washed 4 × 15 min with PBS and 4 × 15 min with distilled water before enhancing with silver (Aurion R-Gent SE-LM, silver enhancement, Electron Microscopy Sciences, www.emsdiasum.com) adjusted from Holgate et al. (1983), see Miller (2006) for further information on immunogold-silver staining. Silver enhancement makes the 10 nm gold particles of the secondary antibody larger and visible in light microscopy: particles show as black dots in bright field microscopy and bright white dots in dark field microscopy. Note that time of exposure to silver enhancement varied with ambient temperature: 10–15 min.

All sections were counterstained with Stevenel's blue before they were mounted in DePex (Electron Microscopy Sciences) and examined by a Leitz Aristoplan light microscope. Stevenel's staining solution is a polychromophore that stains all tissues (del Cerro et al. 1980), which is useful for recognizing cellular structures and thereby areas of interest in bright field microscopy. Moreover, when a low density of antigen makes detection of the target protein challenging in bright field, Stevenel's solution is compatible with dark field imaging as well.

### Western blots

Queen ovaries, abdominal fat body and head fat body tissue were thoroughly washed in a 5 times repeated wash cycle, homogenized and centrifuged in Tris-buffer supplemented with proteinase inhibitors (see above). Hypopharyngeal glands were pooled in replicate from 40 individual nurse bees and 40 individual foragers before washing, homogenization and centrifugation. The supernatants were subjected to one dimensional SDS-electrophoresis using 10% polyacrylamide gels (GeneMate, express gels, 10%, BioExpress, www.bioexpress.com). Separated proteins were transferred to nitrocellulose paper, incubated in polyclonal antibody against vitellogenin (1:25,000), visualized by horse radish peroxidase conjugated secondary antibody (1:150) (Amersham) and developed by chemiluminescence (Western Lightening Chemi Luminescence, Perkin Elmer, www.perkinelmer.com).

## Results

**Figure 6.**
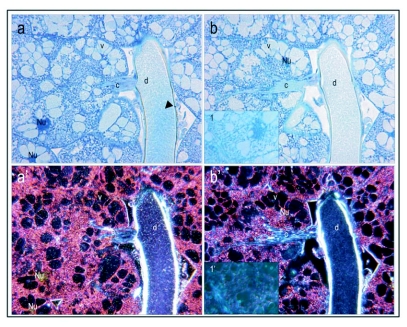
Hypopharyngeal glands at 40 × magnification. At this lower magnification (compare to Fig. 5) the positive labeling is more challenging to detect in bright field mode (a). In dark field mode (a'), however, the stain shows as white spots and is more easily seen in vacuoles (v), the canal (c) and the common duct (d). Blue stained tissue in bright field mode and red and orange colors in dark field mode are contrast staining with Stevenel's blue, a polychromatic dye. Controls were treated with buffer instead of primary antibody and show no positive staining for vitellogenin (b, b'). Corresponding controls treated with preimmune sera are also negative (see inserted box 1 and 1' in b, b', respectively). The gray lining (bright field, a and b) or bright white lining (dark field, a' and b') around the common duct is likely a technical artifact. The lining is not of consistent character within samples, and thus it was not interpreted as positive staining.

**Figure 7.**
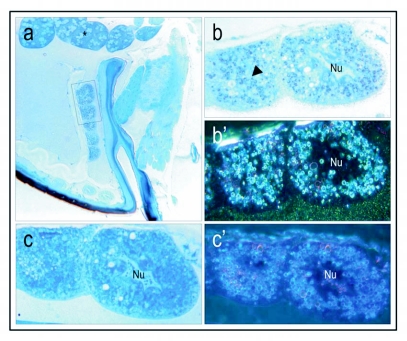
erification of mature vitellogenin protein in the head fat body cells of a nurse bee worker (a, 5 × magnification, see inserted box for the fat cells depicted as close ups at 40 × magnification, b-c). The fat body cells are located in the vicinity of the brood food producing hypopharyngeal glands and thereby close to the brain (a, asterisk). Numerous vitellogenin positive granules are localized in the cells (b, b', see arrow in b). Corresponding controls were treated with preimmune sera and show no positive staining c, c'). Dark field (b') shows positive staining as bright white dots, whereas the corresponding control (c') is negative for vitellogenin.

Honeybee oocyte development is divided into a previtellogenic phase followed by a vitellogenic phase (Engels 1968). The previtellogenic phase primarily takes place in the germarium, which comprises the anterior end of the ovarioles that contain the germ cells and their derivatives (Patricio and Cruz-Landim 2002; Patricio and da Cruz-Landim 2003). The vitellogenic phase occurs in the proximal region of the ovariole where yolk uptake and oocyte growth take place; termed the vitellarium. In worker bees with a queen, the vitellogenic phase is typically blocked, and the ovary as a whole is previtellogenic (Patricio and Cruz-Landim 2002; Patricio and da Cruz-Landim 2003). In accordance with this description, worker bees with a queen had predominantly stage 1 and 2 ovaries (previtellogenic non-activated and -activated ovary, respectively) and did not show positive staining for vitellogenin (Figure 1, stage 1 previtellogenic non-activated ovary, stage 2 not shown). However, in colonies without a queen the majority of the workers had more developed ovaries and vitellogenin was detected in ovaries in stages 3 (vitellogenic ovary with developing oocytes) and 4 (mature ovary with at least one egg) (Figure 2–4). As a general result, differences in vitellogenin staining were not seen between ovaries in the same stage of maturation. This pattern was independent of whether tissues were collected from workers in colonies with or without a queen. Control sections, which were incubated with buffer or preimmune sera instead of primary antibody, were negative for vitellogenin staining (Figure 1–7).

Although worker bees have strongly reduced ovaries (2–16 ovarioles) compared to queens (180–200 ovarioles), the data confirmed that development of worker oocytes corresponds to the process reported for queens (Snodgrass 1956; Velthuis 1970; Engels 1973; Ramamurty and Engels 1977; Fleig 1995). Specifically, in the anterior end of the ovarioles (Figure 2) the activated oogonia divide into one oocyte and several trophocytes (nurse cells) surrounded by a common layer of follicular epithelial cells (Engels 1968). Growing oocytes and clusters of trophocytes alternate from anterior to posterior end and, with continuing development, both the oocyte and the trophocytes increase in volume (Figure 2). The trophocyte subsequently initiates transfer of its contents into the oocyte (Figure 2) (Engels 1968). Additionally, intensely labeled oocytes were present at the posterior end of the ovarioles that seemingly were in the process of disintegration (Figure 2, positive staining shows as black dots in the right ovarioles in panel a and b, controls to the left show no positive staining). Many worker-laid eggs are not viable (Streit et al. 2003), and reabsorption of oocytes is not an uncommon phenomenon (Reginato and Da Cruz-Landim 2002; Patricio and da Cruz-Landim 2003; Tanaka and Hartfelder 2004).

Higher magnification images of vitellogenic oocytes seem to visualize import of vitellogenin (Figure 3, a-b, controls c-d are negative for vitellogenin). Vitellogenin accumulates in the perivitelline space (Figure 3, a), and is subsequently taken up in the oocyte in pinocytotic vesicles that fuse into larger yolk spheres. It is tempting to suggest that at this resolution the staining method supports the two previously reported import mechanisms of vitellogenin: i.e., through and between the follicular cells (Fleig I995), but higher resolution techniques such as transmission electron microscopy would be 1eeded to confirm this. We found that yolk bodies of various sizes can be detected in ovaries of both stage 3 and 4 (Figure 4, a and c). Here, the high density of vitellogenin produces an intense positive staining that shows as dark black in bright field and bright white in dark field. Corresponding controls were treated with preimmune sera instead of primary antibody and show no positive staining for vitellogenin (Figure 4, b, b' and d, d'). After the vitellogenic phase, the vitelline membrane has formed and the yolk bodies have fused into larger vitellogenin-filled spheres (Figure 4, c and c', corresponding controls in d and d'), as described by (Ramamurty and Engels 1977). Here, the less intense positive signal (Figure 4, c and c') could be due to a reduced density of the antigen or crystallization of the protein. Thereby, the vitellogenin dynamics that were identified in worker ovaries are in full accordance with what was previously reported for honeybee queens by means of transport of labeled amino acids from the hemolymph into the ovary (Engels 1973).

After validating the staining method (Fig. 1–4), it was used to test whether vitellogenin protein is present in the hypopharyngeal glands of nurse bees that produce brood food, as hypothesized by Amdam and co-workers (2003). The paired hypopharyngeal glands consist of secretory acini (Costa and Louque 2001; Deseyn and Billen 2005). In each gland, the acini are connected to an axial duct by excretory canals (Deseyn and Billen 2005). The duct ends near the mouthparts of the workers, and via this system the synthesized products of the acini can be transferred by trophallaxis. The vacuoles inside the separate acini, the excretory canals and the common duct contain vitellogenin protein (Figure 5, a and Figure 6, a, a'). Figure 5 a box 1 shows a blow up of the immunogold silver particles that identify vitellogenin. Note that some vacuoles in the hypopharyngeal glands are not positive for vitellogenin staining, as exemplified by the blow up box 2 (Figure 5, a). The amount and localization of antigen in the glands was low and scattered, but persistent within all samples examined (Figure 6). Controls treated with preimmune sera (Figure 5, b) or buffer (Figure 6, b, b') where negative for vitellogenin staining, as required for verification of a specific immunohistochemical method.

These sections also revealed populations of vitellogenin-containing fat body cells in the proximity of the hypopharyngeal glands, and thereby close to the worker brain (Figure 7). Corresponding cells also contain *vitellogenin* mRNA (M. Corona, R. Velarde, S. Remolina, A. Moran-Lauter, K.A. Hughes, G.E. Robinson, unpublished data). The presence of head fat body cells in honeybee workers, queens and drones was documented previously by Snodgrass (1956), and the cells were shown to be similar to the abdominal fat body in structure. The specific dynamics of vitellogenin protein accumulation and spatial localization in the honeybee fat body have not been investigated previously. Yet, similar protein granules with crystalline nuclei have been detected (Koehler 1921; Da Cruz Landim 1985), and such granules are considered to have protein storage functions. Koehler (1921) found albuminoid material in large granules located in the abdominal fat body cells of wintering bees. Snodgrass (1956) subsequently suggested that these albuminoids normally are transformed into brood food, but that they also can accumulate in the fat body as physiological reserves. This hypothesis corresponds to dynamics that more recently were proposed for vitellogenin (Amdam and Omholt 2002). Thus, the finding of vitellogenin in head fat body cells of nurse bees is not surprising, and further shows that our antibody recognizes vitellogenin at one of its native sites of synthesis (Figure 7, a-b, b'; corresponding control treated with preimmune sera instead of primary antibody c,c').

**Figure 8.**
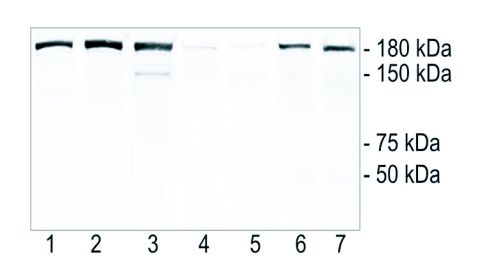
Western blot showing the specificity of the polyclonal antibody. Lane 1: queen abdominal fat body; lane 2: queen head fat body; lane 3: queen ovary; lanes 4 and 5; two independent samples of pooled hypopharyngeal glands from 40 foragers; lanes 6 and 7: two independent samples of pooled hypopharyngeal glands from 40 nurse bees. The antibody recognized a band of 180 kDa that matches the molecular weight of vitellogenin.

To confirm the findings outlined above, Western blot verification was used with selected tissues assumed to be positive or negative for vitellogenin. Samples of ovaries and fat body tissues of queens were included as internal positive controls for the specificity of the antibody because, in comparison with workers, queens are characterized by higher production rates and high titers of vitellogenin throughout adult life (reviewed by Engels et al. 1990). Thoroughly washed nurse bee and forager hypopharyngeal glands (presumed vitellogenin positive and negative, respectively) were run as test samples in duplicate. The polyclonal antibody clearly recognized vitellogenin in queen abdominal fat body, head fat body and ovaries, as well as in the hypopharyngeal glands of nurse bees. The hypopharyngeal glands of foragers were weakly stained (Figure 8, lanes 1–7, respectively). Samples from hypopharyngeal glands, moreover, showed no additional bands. The extra band present in ovary extract is likely due to vitellogenin degradation. This pattern documents that the antibody did not show cross-reactivity with other gland-specific proteins such as the major royal jelly proteins (49–87 kDa (Schmitzovâ et al. 1998)).

## Discussion

A number of publications describe the dynamics of vitellogenin during oocyte maturation in the honeybee (Engels 1972; Engels 1973; Fleig 1995). In the present study ovarian tissue from worker bees with and without a queen were used to develop a visualization method for honeybee vitellogenin. Ovarian tissue is an ideal substrate in the development and evaluation of an immunogold staining procedure. The absence of positive staining in previtellogenic ovaries (Figure 1) and the immunogold cytochemical localization of vitellogenin in vitellogenic tissues (Figure 2–7) demonstrate that the antibody, paired with the presented staining protocol, specifically identifies vitellogenin.

One potential caveat of the method is that worker bees with vitellogenic ovaries have high levels of circulating vitellogenin (Lin et al. 1999). Thus the presence of vitellogenic ovaries may be paired with a higher probability of false positives produced by hemolymph contamination. This can apparently be avoided by implementing an intense washing procedure (see Methods), which strongly reduced the amount of hemolymph present at the time of resin embedding. Detection of vitellogenin in oocytes only at advanced stages of maturation (Fig. 2–4) supports this. Thus, we conclude that the method presented here is both specific to the vitellogenin protein target and also robust against artifactual contamination.

The immunogold localization of vitellogenin in the hypopharyngeal glands of nurse bees (Figure 5–6) is both in accordance and in conflict with previous findings. Amdam and co-workers (2003) identified a vitellogenin receptor in the honeybee worker hypopharyngeal gland that was of the same size as a vitellogenin receptor isolated from queen ovary tissue. They further injected nurse bees with vitellogenin protein that had been synthesized in *vitro* in the presence of C^14^-labeled phenylalanine, and observed that the label was transferred to other colony members. Amdam and co-workers (2003) therefore hypothesized that vitellogenin was utilized in the synthesis of brood food, but that the protein was not transferred directly by trophallaxis. However, a prior study that used immuno-electrophoresis to test for presence of vitellogenin had concluded that vitellogenin is absent from brood food (Rutz and Luscher 1974). Our findings call for a modification of the proposition of Rutz and Luscher (1974), as we demonstrate that vitellogenin is present in the central duct of the hypopharyngeal glands that contains newly synthesized brood food (Figure 5–6). This result is further supported by the Western blot, which shows significant amounts of vitellogenin in hypopharyngeal glands of nurse bees, and very low levels in the hypopharyngeal glands of foragers (Figure 8). The discrepancy between these results and the data of Rutz and Luscher (1974) can be ascribed to differences in sensitivity and specificity of the methods. With use of conventional light microscopy and immuno labeling, discrimination of staining between tissues, cells, and cell compartments is feasible. Immuno-electrophoresis does not offer the same resolution. The insight that vitellogenin protein indeed is present in brood food is relevant to current research, because it has been proposed that evolution of the hypopharyngeal gland transfer-system for vitellogenin, or vitellogenin-derived components, contributed to specialization of the temporal nurse bee- and forager caste of the honeybee (Amdam et al. 2003).

Moreover, recent data indicate that the hypopharyngeal glands, in addition to a role in inter-individual nourishment, can have a storage function similar to a role proposed for the abdominal fat body (Amdam and Omholt 2002). Bees kept in the absence of brood (i.e., diutinus workers or winter bees) have hypertrophied gland acini that may result when secretions of the glands are stored in vesicles (Deseyn and Billen 2005). Fat body tissues, likewise, show accumulation of albuminoidal (protein containing) granules in long-lived workers of colonies without brood (Koehler 1921). Our results show that similar granules in worker head fat body can be vitellogenin positive (Fig. 7). Thus, the data presented here are consistent with a dynamic physiology that allows vitellogenin to be temporary stored in the hypopharyngeal glands and fat body tissues of honeybees, as previously hypothesized by Amdam and Omholt (2002).

The detected presence of vitellogenin protein in worker head fat body is of interest also beyond the context of honeybee storage protein dynamics. Vitellogenin has been hypothesized to affect the allatoregulatory (juvenile hormone controlling) system close to the brain, and thereby the social behavior of worker bees (Amdam and Omholt 2003; Guidugli et al. 2005). As yet the underlying mechanism is not understood, but a connection between vitellogenin and insulin/insulin-like signaling (US), which in turn modulates the allatoregulatory system, is a proposed explanation (Hunt *et al.* 2006; Page and Amdam 2006). In *Drosophila*, US acts in fat body tissue to influence the life history of adults (Giannakou et al. 2004), and the head fat body appears to be of particular importance (Hwangbo et al. 2004). In honeybee workers and queens, US in head fat body is supported by the recent immuno-detection of AmCHICO, the honeybee insulin receptor substrate protein (S.C. Seehuus, F. Wolschin, G.V. Amdam, unpublished data). The interface between head fat body vitellogenin and US, and its possible relationship to behavior, are under study.

Note that during the development of the protocol, it was found that optimal temperature and time for exposure to the silver enhancement step is critical for the immunogold procedure (see Methods). The efficiency of this step is sensitive to ambient temperature, and care must be taken to obtain a particle size that is adequate for observation in light microscopy. Overexposure leads to precipitation of silver that covers the tissue and obscures positive signals. Underexposure leads to small particles that are incompatible with bright field detection at lower magnifications. In this context, it is useful to note that dark field detection is less sensitive to particle size than bright field detection. As a rule, dark field is a valuable tool for detection of low density staining at lower magnifications (16 × 40 ×), whereas bright field detection is more than adequate at higher magnifications (63 x- 100 x) (i.e. Figure 5 vs. Figure 6).

A major advantage of immunohistochemical methods is that they are relatively easy to replicate once a protocol has been established (i.e. Costa and Louque 2001; Bao et al. 2003; Hwangbo et al. 2004). This robustness allows for screening of an array of samples and tissue types, and provides opportunities to pinpoint phenomena for further analysis by other methods. The procedure is sensitive and can provide high-resolution information as exemplified by the indication of membrane transport of vitellogenin (Figure 3). Another advantage is that it can function as a step that precedes sectioning for TEM. The method presented here can favorably be combined with conventional dyes for tissue staining or in situ hybridization. The versatility of the basic immunohistochemical protocol therefore implies that it has broad functionality. Thus, we expect that this protocol for vitellogenin visualization will be useful for future studies aimed at understanding the role and regulation of honeybee vitellogenin in its unconventional target tissues.
